# Angiotensin AT_2_ Receptor Contributes towards Gender Bias in Weight Gain

**DOI:** 10.1371/journal.pone.0048425

**Published:** 2013-01-15

**Authors:** Preethi Samuel, Mohammad Azhar Khan, Sourashish Nag, Tadashi Inagami, Tahir Hussain

**Affiliations:** 1 Department of Pharmacological and Pharmaceutical Sciences, College of Pharmacy, University of Houston, Houston, Texas, United States of America; 2 Department of Biochemistry, Vanderbilt University School of Medicine, Nashville, Tennessee, United States of America; College of Tropical Agriculture and Human Resources, University of Hawaii, United States of America

## Abstract

Obesity is a major disease condition, in turn leading to pathological changes collectively recognized as metabolic syndrome. Recently angiotensin receptor AT_2_R has been associated negatively with body weight (BW) gain in male mice. However, the gender differences in AT_2_R and BW changes have not been studied. To understand the gender based role of AT_2_R involving BW changes, we fed male and female wild type (WT) and AT_2_R knock out (AT_2_KO) mice with C57BL6 background with high fat diet (HFD) for 16 weeks. The male AT_2_KO had higher HFD calorie intake (WT: 1280±80; AT_2_KO:1680±80 kcal) but gained less BW compared with the WT (WT: 13; AT_2_KO: 6 g). Contrary to the male animals, the female AT_2_KO mice with equivalent caloric intake (WT: 1424±48; AT_2_KO:1456±80 kcal) gained significantly more BW than the WT mice (WT: 9 g; AT_2_KO: 15 g). The male AT_2_KO on HFD displayed lower plasma insulin level, less impaired glucose tolerance (GT), and higher plasma T3 compared with WT males on HFD; whereas the female AT_2_KO mice on HFD showed elevated levels of plasma insulin, more impaired GT, lower plasma T3 and higher free fatty acid and hepatic triglycerides compared with WT females on HFD. Interestingly, compared with WT, AT_2_KO female mice had significantly lower estrogen, which was further reduced by HFD. These results suggest that AT_2_R in female mice via potentially regulating estrogen may have protective role against BW gain and impaired glucose tolerance and lipid metabolism.

## Introduction

Obesity by itself is a major disease condition, which, in turn, leads to a host of associated pathological changes collectively recognized as metabolic syndrome. [1] Renin angiotensin system (RAS) in addition to playing a critical role in regulating blood pressure (BP) and maintaining electrolyte balance has been reported to contribute towards the initiation and progression of metabolic syndrome.[2]–[4] Actions of angiotensin II, the major peptide hormone of RAS, are mediated via its two receptors namely AT_1_R and AT_2_R. [5] Studies suggest that absence or blockade of AT_1a_R in mice results in resistance of diet-induced obesity, improvement of glucose tolerance and insulin sensitivity, and protection against some traits of metabolic syndrome. [6], [7] Studies using male AT_2_KO mice have shown a negative relationship between AT_2_R and body weight gain, implying that absence of AT_2_R is associated with lower body weight gain.[8]–[11] However, these studies have only included male mice.

Gender based differences in both humans and animal models are known to exist with regards to BP regulation, progression of renal damage, inflammation, weight gain and obesity.[12]–[14] These differences have primarily been attributed to changes and alterations by sex hormones. [9] Estrogen in particular has been implicated in rendering female gender specific protection of physiological changes. [15] Further, many of these selective advantages such as glucose intolerance and vasodilatation in females are potentially brought about by interaction of estrogen with other hormones and receptors such as insulin and recently with AT_2_R. [16], [17] Recent evidence indicates a positive role of AT_2_R in physiological changes by an independent action as well as by influencing interactions with other components of RAS system including AT_1_R and angiotensin I converting enzyme 2 (ACE2). [15] While the protective role of AT_2_R in lowering BP in females has been recently reported, [18] the role of AT_2_R in gender based changes to body weight is not known. While obesity is described as an overall disorder of energy balance resulting from increase in food intake and/or reduced energy expenditure, it is also symptomatically characterized by impaired glucose tolerance, hyperinsulinemia and enhanced plasma free fatty acids (FFA) that in turn contributes further towards insulin resistance.[19]–[21] The present study was designed to understand the differential role of AT_2_R in body weight changes and metabolic parameters between male and female mice. We found that at the end of 16 weeks of high fat diet (HFD), absence of AT_2_R had opposite effects on the body weight gain, glucose tolerance, and plasma insulin of males and females; and these changes were inversely related to several factors including changes in the estrogen levels, and physiological role of AT_2_R in males and females.

## Methods and Procedures

### Animals

C57BL6 breeder pairs (11–12 weeks of age) were obtained from Harlan (Indianapolis, IN) to create F1 WT mice population. The AT_2_ knockout females and males with C57BL6 background were used to breed AT_2_R knockout mice. [22] The F1 generation (3–4 weeks old) of both WT (28 males, 27 females) and AT_2_KO mice (18 male; 26 female) were placed either on normal diet (ND with % kcal 29∶15∶56 protein:fat:carbohydrate) or high-fat diet (HFD with adjusted % kcal 18∶60∶21protein:fat:carbohydrate) for 16 weeks. The mice were housed in the University of Houston animal care facility and maintained under a 12-hr light/dark cycle**.** The ND/HFD and water were provided *ad libitum* throughout the experiment period. The Institutional Animal Care and Use Committee approved the animal experimental protocols at the University of Houston, Houston, TX.

### Food Intake and Body Weight

To determine the direct impact of food intake differing in caloric content on body weight, we measured the food intake throughout the length of the study. Since mice thrive in a social group for a long-term study like the present, with the exception of a few aggressive male mice, the mice was housed in social groups of 2–4 animals in each cage. Weekly 100 g of food in the form of pallet was placed in the food slot of the mice cages. At the end of each week the remaining food, including any fallen bits, was weighed on an electronic weighing scale (Denver instrument P-602) and the amount subtracted from the initial 100 g to account for the food consumed. The total intake of each cage was then divided by the number of mice in the cage. Three days prior to sacrifice, the mice were placed individually in metabolic cages to record the food consumption, water intake, urine output and other parameters for single mouse. The food intake in the different groups was calculated in terms of calorie consumed. We measured the change in body weight in the different groups of mice to assess the effect of the diet and treatment on the body weight. Body weight in the different groups also was measured every week for each animal on a triple beam veterinary scale (Ohaus 730-00 veterinary balance) and measured as change in body weight over basal, which was weight measured at the start of the dietary regimen.

### Glucose Tolerance Test (GTT)

While obesity is described as an overall disorder of energy balance resulting from increase in food intake and/or reduced energy expenditure, it is also symptomatically characterized by impaired glucose metabolism which further contributes towards glucose intolerance.[19]–[21] To study the effect of HFD feeding on carbohydrate metabolism, a standard GTT was performed in fasting animals. Briefly, in week 16 of the dietary regimen, the mice of different groups were fasted for 6 hours and given 25 mmol of glucose through intraperitoneal injection. The blood glucose was measured by pricking the tail of conscious animals at 0, 15, 30, 60, 120 and 180 minutes using a glucometer.

### Plasma and Urine Collection, and Gonadal Fat Removal

Prior to the termination of experiment all animals were individually housed in mouse metabolic cages for 3 consecutive days for urine collection. At the end of 16-weeks treatment, the mice were euthanized by cervical dislocation under anesthesia following 6 hours of fasting. Plasma was collected and stored at −80°C until further use. Gonadal fat depot is the largest dissectable fat pad in rodents and accounts for 30% of all removable fat whose mass has been used as an index of obesity. Therefore, to correlate changes in body weight with the fat content, we measured the mass of gonadal fat depot in the different groups. Paired-gonadal adipose depot (surrounding the epididymus and testes in males, and uterus and ovaries in females) were removed, pat dry on tissue paper and weighed.

### Enzyme-linked Immunosorbent Assay (ELISA) for Plasma Insulin and Tri-iodothyronine (T3)

Hyperinsulinemia is an index of insulin resistance which is known to correlate with obesity. Furthermore, AT_2_R deficiency has been shown to prevent diet-induced insulin resistance in male mice. [8] Therefore, we measured the plasma insulin levels to assess gender based role of AT_2_R on insulin resistance in HFD-induced obesity. Plasma insulin and T3 levels in different groups were determined using ELISA kit as per the manufacturer’s instructions. For insulin, 5 µl of blank, standard, and sample with 95 µl of sample diluent was added in duplicates to appropriate wells. The plate was incubated for 2 hours at room temperature (RT). The wells were washed 5 times and 100 µl of anti-insulin enzyme conjugate was added to each well and incubated for 30 min at room temperature. The wells were washed 7 times, 100 µl of enzyme substrate was added and incubated in dark for 40 min. 100 µl of stop solution was added to stop the reaction and absorbance was measured on a micro-plate reader (BMG LABTECH, Inc. Cary, NC) at A_450_ and subtracted from A_630_ values. Since obesity is associated with metabolic/physiological changes either contributed by or as a result of alterations in the endocrine system, we measured a critical metabolic hormone T3, whose plasma level is known to positively correlate with metabolic rate. [23] For T3 levels, the microplate-wells were formatted and 25 µl of appropriate serum reference, control or sample added into assigned wells in duplicates. 100 µl of working T3-enzyme conjugate solution was added to all wells and incubated for 60 min at room temperature with shaking. Following incubation the wells were washed 3 times and 100 µl of TMB substrate solution was added to all wells and incubated at room temperature for 15 min. The reaction was stopped by adding 50 µl of stop solution. The absorbance was read at A_450_ within 15 min on the micro-plate reader.

### Free Fatty Acid (FFA) and Triglyceride (TG) Levels

Obesity is known to be associated with enhanced plasma FFA, which in turn contributes towards insulin resistance.[19]–[21] Plasma FFA level was measured by colorimetric method using FFA quantification kit. For hepatic TG, livers were dissected and snap-frozen in liquid nitrogen and stored at −80°C. In brief, lipids were extracted by homogenizing 25 mg liver tissue in 1 ml 5% Triton-X100 in water, then slowly heated to 80°C in water bath for 5 min. The samples were cooled down and again heated to solubilize all triglycerides into solution. The samples were centrifuged for 5 min and supernatants were diluted 10 fold with dH_2_O for quantification.

### Enzyme Immune Assay (EIA) for Urinary 17-β-estradiol

Low levels of estrogen could lead to accumulation of fat mass and development of obesity and hyperlipidemia in females. [9], [24] Estrogen also has been shown to have a positive interaction with AT_2_R. [25], [26] In light of this information, we measured urinary estradiol (E2) levels in both male and female animals to investigate a correlation/explanation of body weight changes with estrogen in both genders. Extraction from the urine samples was done by mixing the individual samples with methylene chloride and allowing layers to separate. The extraction was repeated twice and methylene chloride layer was separated followed by evaporation at 30°C. The residue was dissolved in 0.5 ml of EIA buffer. Estradiol level in different groups of female mice was determined by taking 50 µl of appropriate blank, standard or sample into assigned wells in duplicates. After adding estradiol tracer and EIA antiserum to appropriate wells, the plate was incubated at room temperature for 1 hour. The plate was developed by adding 200 µl of Ellman’s reagent to each well and incubating in dark on orbital shaker for 90 min. The absorbance was read at A_410_ on the micro-plate reader. The data was calculated for 17-β-estradiol excreted in 24 hr urine.

### Chemicals

The HFD feed Teklad custom research diet (catalogue # TD.06414) with adjusted calorie diet (60/Fat) containing 18.4% protein, 21.3% carbohydrate and 60.3% fat, and normal diet 7022 with isocaloric 29% protein, 56% carbohydrate and 15% fat were purchased from Harlan, Indianapolis, IN. Both diets contained similar mineral mix AIN-93G-MX (94046) and vitamin mix AIN93-VX (94047). The Ultra-Sensitive mouse ELISA kit catalog # 90080 from Crystal Chem. Inc. Downers Grove, IL was used for plasma insulin. The Mouse/Rat T3 total, ELISA kit catalog # T3043T-100 from CALBIOTECH, Spring Valley, CA was used for T3. Plasma free fatty acid levels was measured by free fatty acid quantification kit (Abcam-cat# ab65341, USA). Hepatic TG was analyzed using the triglyceride quantification kit (Abcam-cat# ab65336, USA). The Estradiol EIA kit 96 solid wells, EIA kit catalog # 582251 from Cayman Chemical, Ann Arbor, MI was used for estradiol.

### Statistics

Data are presented as mean±SEM. The data were analyzed using GraphPad Prism 5 (GraphPad software, San Diego, CA) and subjected to one-way ANOVA with Newman-Keuls post hoc test and Student’ unpaired *t* test. A *p* value of less than 0.05 was considered statistically significant. Power analysis was performed on groups with higher internal variation to determine statistical significance.

## Results

### Food Intake

**Figure 1:**
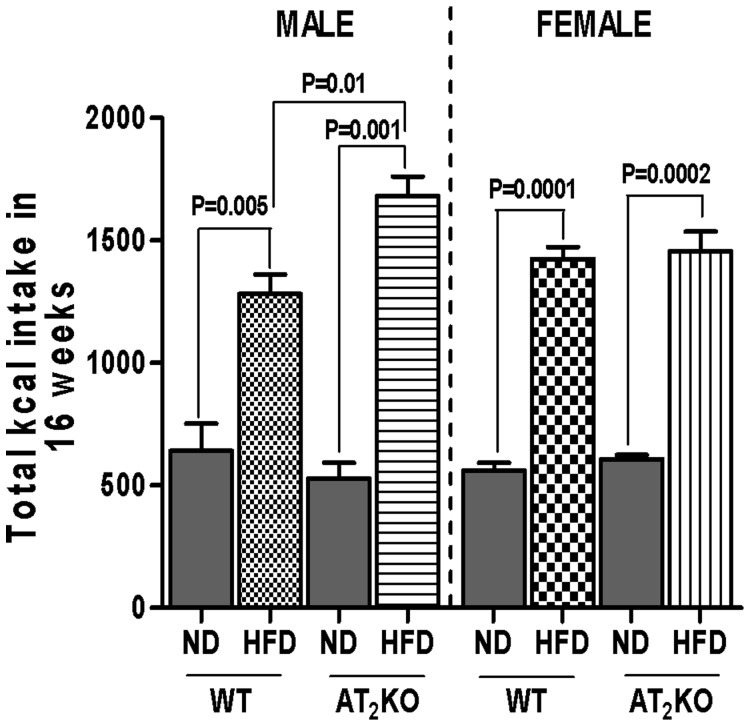
Total calorie intake consumed over 16 weeks by the different groups of mice on normal diet (ND) and high fat diet (HFD). Weekly 100 g of food in the form of pallet was placed in the food slot of the mice cages. At the end of the week, remaining food was weighed and the amount subtracted from the initial 100 g to account for the food consumed. The food intake in the different groups was calculated in terms of calorie consumed. Data analyzed using one-way ANOVA with Newman-keuls post hoc test and Student *t* test (p<0.05); n for males (WTND = 13; WTHFD = 15; AT_2_KOND = 7; AT_2_KOHFD = 11); n for females (WTND = 7; WTHFD = 20; AT_2_KOND = 10; AT_2_ KOHFD = 17).

The food intake was calculated in terms of total kilo calorie (kcal) consumed over 16 weeks. We found no significant difference in the kcal consumption of ND among the males and females of either WT or AT_2_KO (Figure 1). However, the kcal intake was significantly higher in the HFD-fed group compared to their respective ND-fed controls (for males WT P = 0.005; AT_2_RKO P = 0.001 and for females WT P = 0.0001; AT_2_RKO P = 0.0002). Furthermore, the AT_2_KO male mice on HFD had significantly higher kcal consumption compared with WT males on HFD (P = 0.01) (Figure 1).

### Body Weight

**Figure 2:**
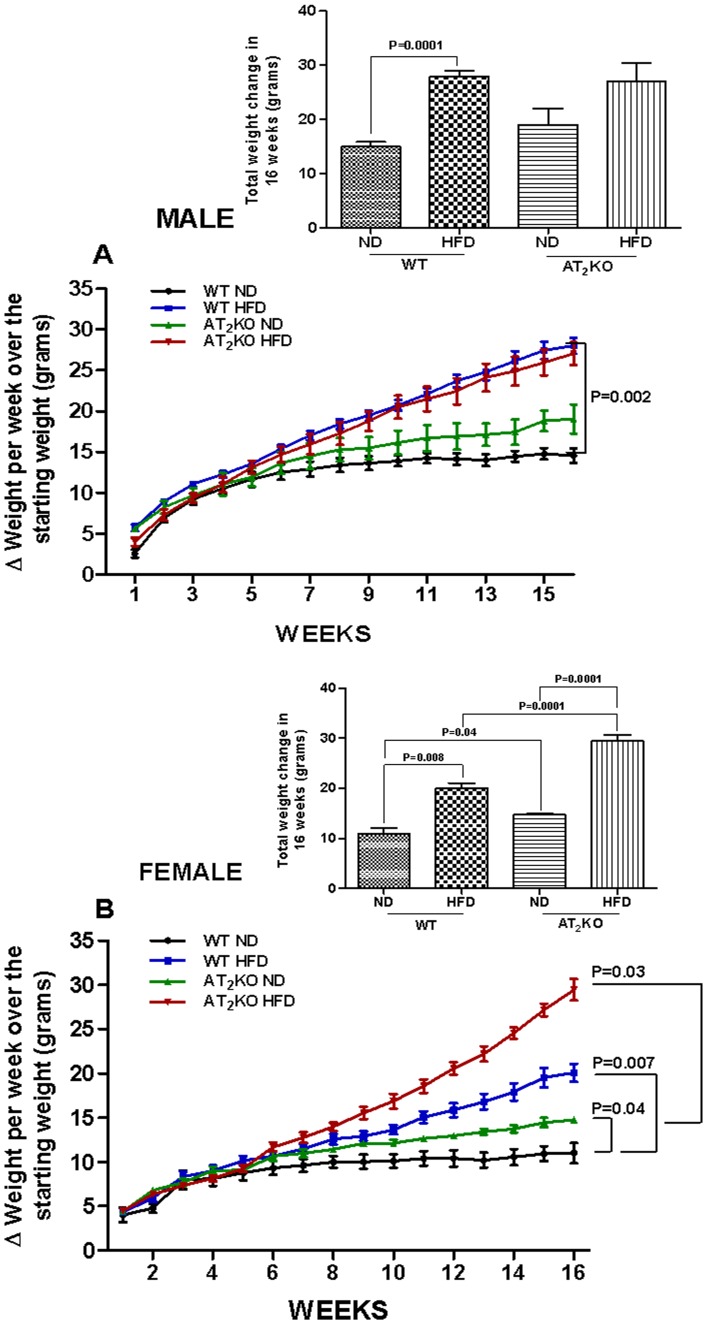
Body weight gain in AT2KO mice on high fat diet. Cumulative body weight change (per week) in normal diet (ND) or high fat diet (HFD) fed WT and AT_2_KO (A) male and (B) female mice. *Insets*- Total body weight change in 16 weeks. Body weight in the different groups was measured every week for each animal on a triple beam veterinary scale and measured as change in body weight over basal, which was weight measured at the start of the dietary regimen. Data analyzed using one-way ANOVA with Newman-keuls post hoc test and Student *t* test (p<0.05); n for males (WTND = 13; WTHFD = 15; AT_2_KOND = 7; AT_2_KOHFD = 11); n for females (WTND = 7; WTHFD = 20; AT_2_KOND = 10; AT_2_ KOHFD = 17).

Weight gain pattern in male and female AT_2_KO mice on HFD clearly was opposite. In males HFD had a similar effect on body weight gain in both WT and AT_2_KO. In WT starting from comparable body weights, HFD caused significant increase (28±1 g) compared with ND (15±0.8 g) (P = 0.002) with net difference in 13 g. On the other hand, the difference between AT_2_KO males on HFD (27±0.3 g) and AT_2_KO on ND (19±3 g) was attenuated to 8 g. This difference in AT_2_KO weight on ND and HFD, in part could be attributed to increased weight in AT_2_KO on ND. Contrary to the males, AT_2_KO female mice on ND (15±0.3 g) as well as on HFD (30±1 g) gained body weight significantly greater than WT on ND (11±1.2g) and WT on HFD (20±1 g), respectively (Figure 2B). We also examined the slope of weight gain curves over 16 weeks. Following are the slopes: males (WT-ND 0.23±0.03; WT-HFD 1.31±0.02) (P = 0.0001), (AT_2_KO-ND 0.55±0.04; AT_2_KO-HFD 1.27±0.02) (P = 0.0001); females (WT-ND 0.14±0.0; WT-HFD 0.95±0.03) (P = 0.0001); (AT_2_KO-ND 0.40±0.01; AT_2_KO-HFD 1.77±0.07) (P = 0.0001). The data suggest that HFD caused changes in the slope of weight gain in both WT and AT_2_KO males and females. However, the slope of weight gains in WT males on HFD and AT_2_KO males on HFD are similar, but there was a significant increase in AT_2_KO females on HFD compared WT females on HFD. To determine the overall pattern of weight changes in various groups, we determined area under the curve (AUC) of weight gain over 16 weeks. The AUC data in males are: WT-ND 151±1.6 vs. WT-HFD 236±2.7 (P = 0.001) and AT_2_KO-ND 177±1.8 vs. AT_2_KO-HFD 226±2.4 and in females are: WT-ND 102±1.3 vs. WT-HFD 151±1.6 and AT_2_KO-ND 127±1.3 vs. AT_2_KO-HFD 193±2) (P = 0.001). The data clearly suggests that changes in AUC pattern in males and females are opposite and follow the end point weight gain data in males and females after 16-weeks of dietary regimen. In males, the AUC increase (49 units) in AT_2_KO HFD over ND was lesser than the AUC increase (85 units) in WT HFD over ND. Contrarily, in females the AUC increase (66 units) in AT2KO HFD over ND was greater than the AUC increase (49 units) in WT HFD over ND.

### Gonadal Adipose Depot Mass

**Figure 3:**
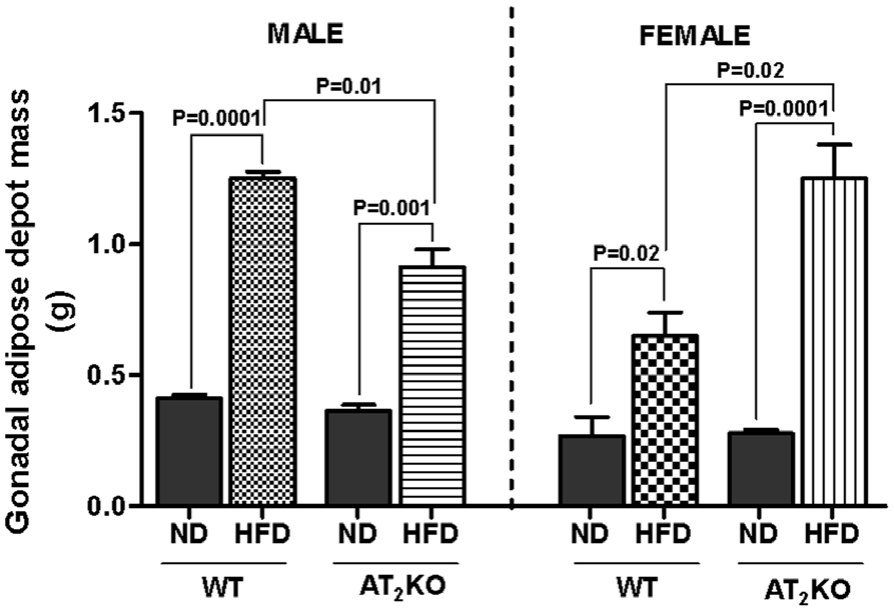
Gonadal adipose depot mass in male and female WT or AT_2_KO mice on normal diet (ND) or high fat diet (HFD). At the end of 16-weeks treatment, following sacrifice, paired gonadal adipose depot (surrounding the epididymus and testes in males, and uterus and ovaries in females), were removed, pat dry on tissue paper and weighed. Data were analyzed using one-way ANOVA with Newman-keuls post hoc test and Student *t* test (p<0.05); n for males (WTND = 4; WTHFD = 3; AT_2_KOND = 3; AT_2_KOHFD = 4); n for females (WTND = 4; WTHFD = 3; AT_2_KOND = 6; AT_2_KOHFD = 3).

As shown in figure 3, in WT males, HFD significantly increased the epididymal fat pad weight (1.2±0.02 g) compared with ND (0.4±0.01 g) (P = 0.0001). The AT_2_KO males on HFD gained (0.9±0.06 g) significantly higher fat mass compared with AT_2_KO mice on ND (P = 0.001); but there was a significant decrease in epididymal fat weight between male AT_2_KO and WT on HFD (P = 0.01). On the other hand, AT_2_KO female mice on HFD (1.2±0.13 g) gained fat weight significantly greater than WT on HFD (0.6±0.09 g) (P = 0.02).

### Glucose Tolerance Test (GTT)

**Figure 4:**
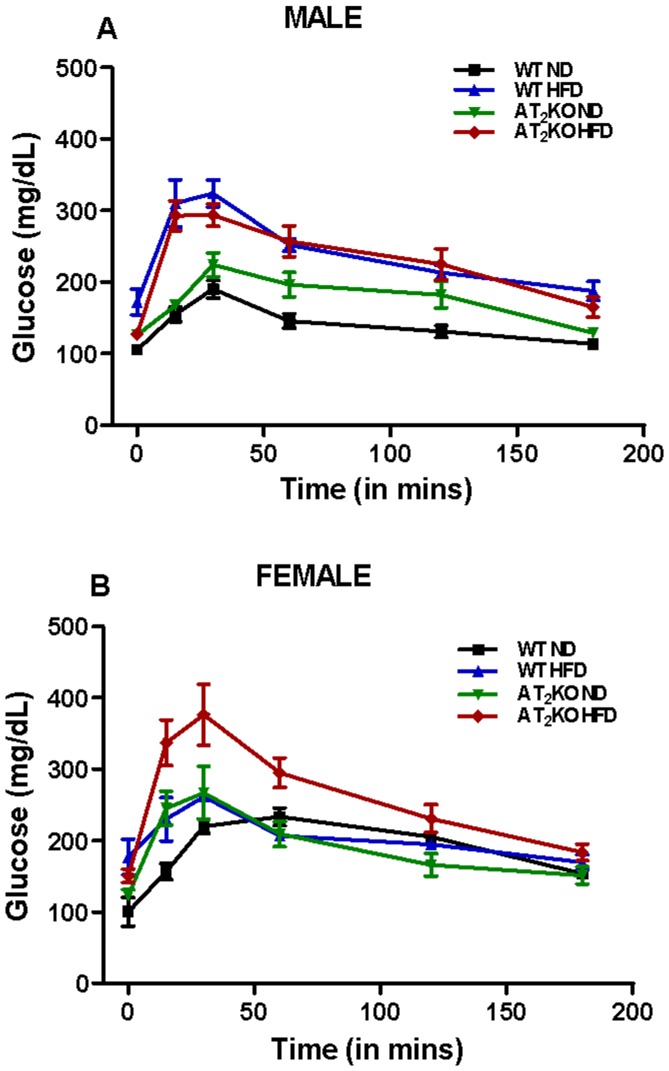
Effect of high fat diet on glucose tolerance in AT2KO mice. Glucose tolerance test (GTT) curves in (A) male and (B) female WT or AT_2_KO mice on normal diet (ND) or high fat diet (HFD). (C) Peak values and (D) area under curve (AUC) of GTT male and female WT and AT_2_KO on ND or HFD. In week 16 of the treatment, the mice of different groups were fasted for 6 hours and given 25 mmol of glucose through IP injection. The blood glucose was measured by pricking the tail of conscious animals at 0, 15, 30, 60, 120 and 180 minutes using a glucometer. Data were analyzed using one-way ANOVA with Newman-keuls post hoc test and Student *t* test (p<0.05); n for males (WTND = 5; WTHFD = 4; AT_2_KOND = 4; AT_2_KOHFD = 5); n for females (WTND = 4; WTHFD = 4; AT_2_KOND = 5; AT_2_KOHFD = 6).

Figures 4A and 4B represent the blood glucose over 3 hours period after glucose infusion in male and female mice subjected to 6 hour prior fasting. These blood glucose values measured at the different time points were used to construct glucose curves that were used for calculating GT curve peak and area under the curve (AUC) (Figure 4C &D). The GT curve peak in HFD fed WT (344±27 mg/dL) and AT_2_KO males (307±15 mg/dL) was significantly higher compared with ND fed WT (217±22 mg/dL) (P* = *0.01) and AT_2_KO (224±17 mg/dL), respectively (Figure 4C). Both the curve peak and AUC in HFD fed AT_2_KO (curve peak 307±15 mg/dL; AUC 37560±1942) were modestly lower than WT (curve peak 344±27 mg/dL; AUC 42970±1615); however, the decrease was not statistically significant.

On the other hand in females, the GT curve peak in HFD fed WT (269±11 mg/dL) and AT_2_KO (438±25 mg/dL) was significantly higher compared with their respective ND-fed control groups, WT (235±5 mg/dL) (*p*<0.04) and AT_2_KO (304±32 mg/dL) (P* = *0.02) (Figure 4C). The AUC followed similar pattern for the AT_2_KO group with the AUC of HFD fed (49550±3236) being significantly greater than the ND fed (33080±1269) (P = 0.004) (Figure 4D). However, the curve peak as well as the AUC in AT_2_KO female mice (Curve peak 438±25 mg/dL; AUC 49550±3236) in response to HFD was profoundly greater than the increase detected in WT females on HFD (Curve peak 269±11 mg/dL; AUC 36740±1113) (P* = *0.003 and P* = *0.03) respectively) (Figure 4C, D).

### Plasma Insulin

**Figure 5:**
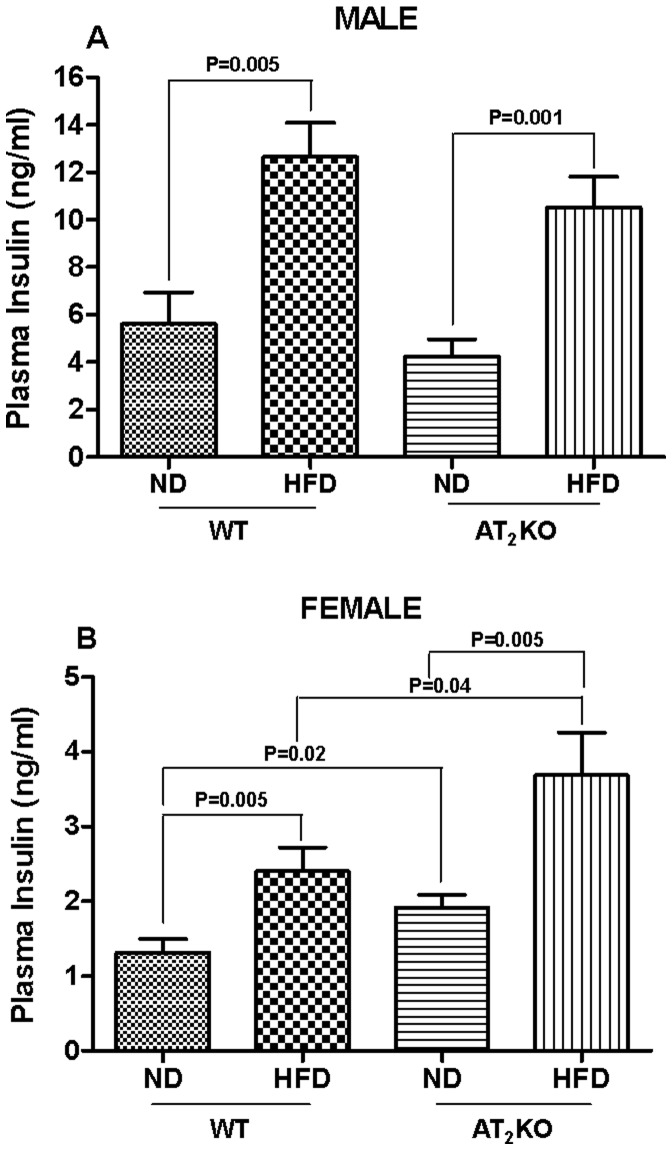
Effect of high fat diet on hyperinsulinemia in AT2KO mice. Plasma insulin levels in (A) male and (B) female WT and AT_2_KO fed on normal diet (ND) and high fat diet (HFD). Plasma insulin levels in different groups were determined using ELISA kit as per the manufacturer’s instructions. Data were analyzed using one-way ANOVA with Newman-keuls post hoc test and Student *t* test (p<0.05); n for males (WTND = 20; WTHFD = 8; AT_2_KOND = 6; AT_2_KOHFD = 6); n for females (WTND = 19; WTHFD = 20; AT_2_KOND = 16; AT_2_KOHFD = 16).

All HFD fed groups, WT and AT_2_KO males and females, exhibited greater plasma insulin levels compared with their respective ND control groups (P = 0.01- P = 0.001). However, plasma insulin in AT_2_KO males on HFD (11±1.3 ng/ml) was modestly lower than WT on HFD (13±1.4 ng/ml) (Figure 5A). Contrary to the males, plasma insulin in AT_2_KO females on HFD (3.7±0.6 ng/ml) was significantly higher than WT on HFD (2.4±0.3 ng/ml) (P = 0.04) (Figure 5B). Also, AT_2_KO females on ND (1.9±0.2 ng/ml) exhibited modestly but significantly higher plasma insulin compared with WT on ND (1.3±0.2 ng/ml) (P = 0.02) (Figure 5B).

### Plasma T3

**Figure 6:**
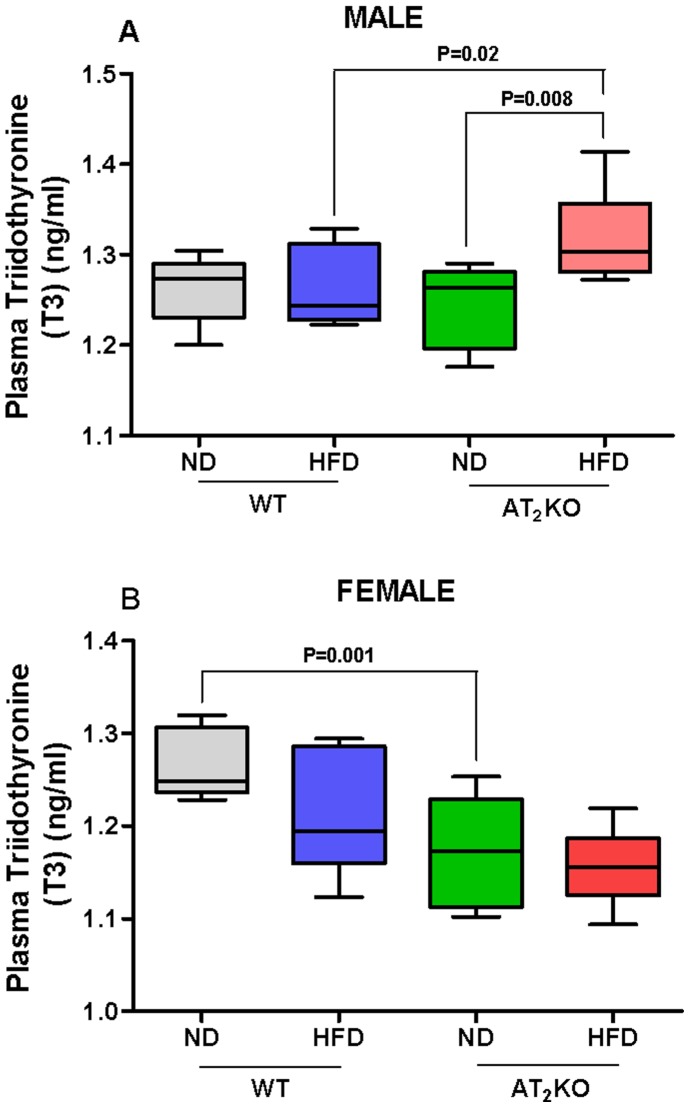
Effect of high fat diet on plasma T3 in AT2KO mice. Plasma T3 levels in (A) male and (B) female of WT or AT_2_KO mice normal diet (ND) and high fat diet (HFD). Plasma T3 levels in different groups were determined using enzyme-linked immunosorbent assay (ELISA) kit as per the manufacturer’s instructions. Data analyzed using one-way ANOVA with Newman-keuls post hoc test and Student *t* test (p<0.05); n for males (WTND = 8; WTHFD = 8; AT_2_KOND = 8; AT_2_KOHFD = 8); n for females (WTND = 8; WTHFD = 8; AT_2_KOND = 8; AT_2_KOHFD = 8).

The HFD had no effect on plasma T3 levels in WT males (ND: 1.26±0.01 ng/ml; HFD: 1.26±0.01 ng/ml) but caused a modest increase in T3 in the AT_2_KO males (ND: 1.27±0.01 ng/ml; HFD: 1.32±0.02 ng/ml) (P = 0.008) (Figure 6A). On the other hand, the plasma T3 levels were not affected by HFD in female mice, WT or AT_2_KO, compared with females on ND (Figure 6B). However, AT_2_KO females on ND (1.17±0.02 ng/ml) exhibited lower plasma T3 levels compared with WT females on ND (1.26±0.01 ng/ml) (P = 0.001) (Figure 6B).

### Plasma Free Fatty Acids (FFA) and Hepatic Triglycerides (TG)

**Figure 7:**
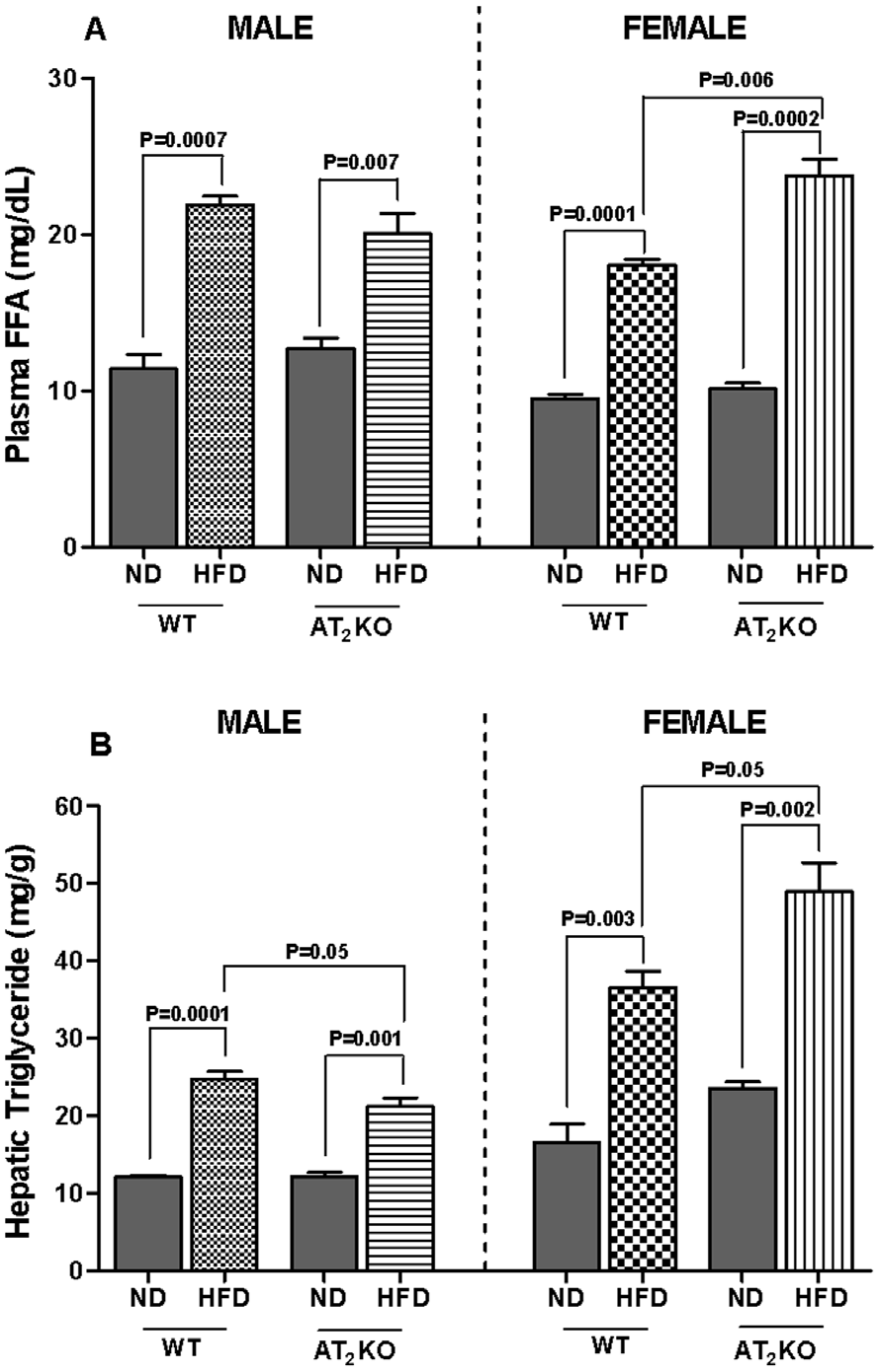
Effect of high fat diet on plasma hepatic free fatty acids and hepatic triglycerides in AT2KO mice. Plasma FFA (A) and Hepatic triglycerides (B) levels in male and female WT or AT_2_KO mice either on normal diet (ND) or high fat diet (HFD). Plasma FFA level was measured by colorimetric method using FFA quantification kit. Hepatic TG level was measured by colorimetric method using TG quantification kit. Data analyzed using one-way ANOVA with Newman-keuls post hoc test and Student *t* test (p<0.05). In plasma FFA, n for males (WTND = 3; WTHFD = 3; AT_2_KOND = 3; AT_2_KOHFD = 3); n for females (WTND = 7; WTHFD = 3; AT_2_KOND = 3; AT_2_KOHFD = 3). For hepatic triglycerides, n for males (WTND = 4; WTHFD = 3; AT_2_KOND = 4; AT_2_KOHFD = 3); n for females (WTND = 3; WTHFD = 3; AT_2_KOND = 3; AT_2_KOHFD = 4).

The HFD caused a significant increase in plasma FFA (Figure 7A) and hepatic TG (Figure 7B) in both the WT and AT_2_KO males. AT_2_KO males on HFD had significantly (P = 0.05) lower TG (21.2±1 mg/g tissue) compared with HFD fed WT (TG: 25±0.1 mg/g tissue). The FFA in AT_2_KO males on HFD (20.1±1.3 mg/dL) were modestly, but non-significantly, reduced compared with HFD WT (22±0.6 mg/dL). In females, both WT and AT_2_KO on HFD had elevated plasma FFA (Figure 7A) and hepatic TG (Figure 7B) compared with their respective controls; but the increase in FFA and TG was much greater in AT_2_KO females (FFA: 24±1 mg/dL(P = 0.0002); TG: 49±4 mg/g (P = 0.002) compared with WT females on HFD (FFA: 18±0.4 mg/dL (P = 0.0001); TG: 37±2 mg/g (P = 0.003).

### Urinary 17-β-estradiol

**Figure 8:**
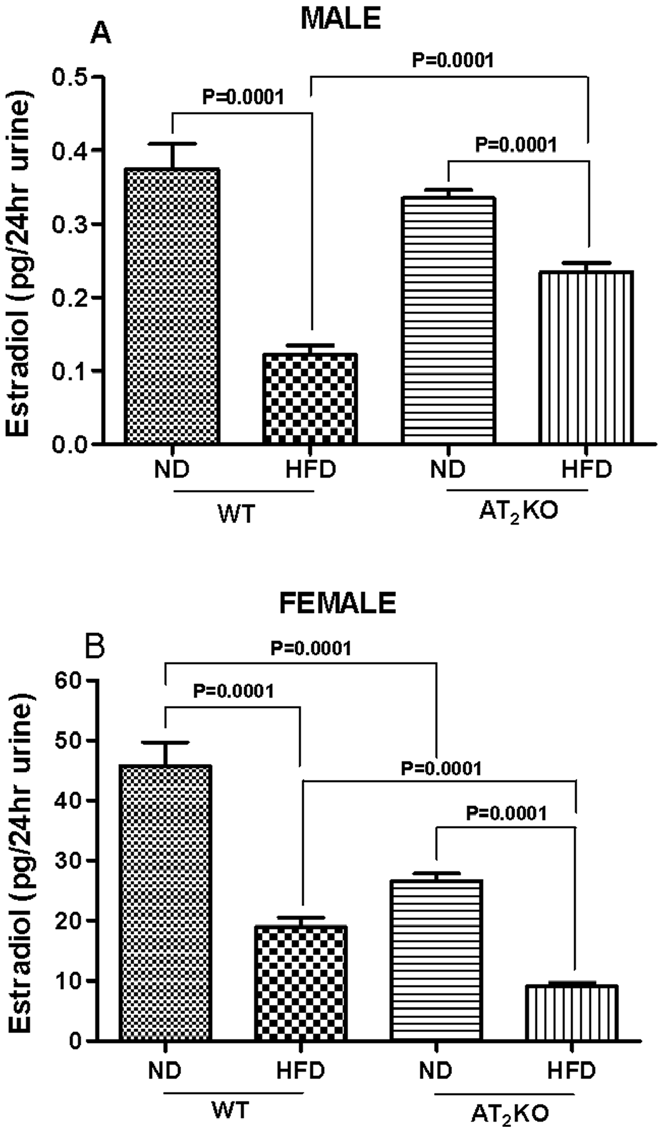
Effect of high fat diet on urinary estradiol in AT2KO mice. Urinary estradiol levels of (A) male and (B) female WT or AT_2_KO female mice. The urinary 17-β-estradiol was measured using a commercial EIA (Enzyme immune assay) kit as per manufacturer’s instruction. Data analyzed using one-way ANOVA with Newman-keuls post hoc test and Student *t* test (p<0.05); n for males (WTND = 6; WTHFD = 6; AT_2_KOND = 6; AT_2_KOHFD = 6); n for females (WTND = 6; WTHFD = 8; AT_2_KOND = 13; AT_2_KOHFD = 13).

The urinary estradiol (E2) level in the WT female mice on HFD (19±1.5 ng/24 hr) was lower compared with the WT females on ND (46±4 ng/24 hr). Interestingly, the urinary E2 level in the AT_2_KO female mice on ND (27±1.3 ng/24 hr) was significantly lower than WT females (P = 0.0001) and was further reduced by HFD (9±0.6 ng/24 hr) (P = 0.0001) (Figure 8B). The urinary E2, even though a predominant female hormone, also was measured in male mice (Figure 8A). The E2 levels in males are in a much lower ranges. However, the urinary E2 levels in the WT male mice on HFD (0.12±0.01 ng/24hr) was significantly lower compared with the WT males on ND (0.37±0.03 ng/24 hr) (P = 0.0001). Similarly, the E2 levels in the AT_2_KO male mice on HFD (0.23±0.01 ng/24 hr) was significantly lower compared with AT_2_KO males fed on ND (0.33±0.01 ng/24 hr) (P = 0.0001), but was significantly greater than WT on HFD (P = 0.0001) (Figure 8A).

## Discussion

In the present study, we found that AT_2_KO males on HFD had lesser body weight gain, lower gonadal adipose depot weight, lower levels of plasma insulin less impaired glucose tolerance (GT), higher plasma T3 and higher urinary estrogen levels, compared with HFD fed WT males. In contrast to the males, AT_2_KO females on HFD had significantly higher body weight, higher gonadal adipose depot weight, elevated levels of plasma insulin, more impaired GT, lower plasma T3 and estrogen levels, compared to HFD fed WT female mice. Additionally, the urinary E2 in AT_2_KO females on ND was lower compared with WT on ND.

Excessive weight gain or obesity is generally associated with hyperinsulinemia, impaired glucose tolerance and blood pressure increase [19]–[21]. Moreover, obesity and associated metabolic/physiological changes are contributed by or result in the alterations in various hormone systems, including thyroid hormone. [23] In the present study, we observed that plasma T3 was increased in the AT_2_KO males following 16 wk of HFD treatment (Figure 6). However, in AT_2_KO females T3 levels were significantly lower compared to the WT. Although, we have not measured metabolic rate or related parameters, changes in plasma T3 AT_2_KO mice indicates a possible slowing down of basal metabolic rate in AT_2_KO female mice. Interestingly, the T3 levels of ND fed AT_2_KO female were not different from AT_2_KO on HFD indicating the basal levels of T3 in AT_2_KO to be inherently lower and not subject to changes by dietary manipulation. However, the changes in T3 levels are very modest; whether such changes in T3 are able to affect the metabolic rate is not clear.

The weight gain pattern of AT_2_KO males in our study concurs with other reports showing that AT_2_KO mice in response to HFD had a lower rate of weight gain compared to the WT mice. [8], [11] Moreover, these studies showed that inhibition of AT_2_R improved insulin sensitivity and caused fat loss, by influencing insulin signaling pathway and control of adipose tissue metabolism. On the other hand, although there is a study linking AT_2_R A/C^3123^ polymorphism with increased body mass index in Japanese women, [27] ours is the first study demonstrating that lack of AT_2_R results in the greater weight gain in female animals placed on HFD.

In mice of the eight major adipose depots four are present in the abdominal cavity. Gonadal depot present in the abdominal cavity is the largest dissectable adipose depot, [28] consisting of 30% dissectable fat. [29] A cluster of metabolic disorders and diseases have been closely associated with abdominal obesity in humans. [30] The changes in body weight gain in the present study are associated with parallel changes in the gonadal adipose depot in both WT and AT_2_KO mice on HFD (Figure 3). The changes in the fat mass suggest that AT_2_R differentially affects fat metabolism in males and females and contributes to body weight gain in response to HFD.

In order to explain the gender based role of AT_2_R in weight gain, we measured urinary estrogen, which serves as an index of endogenous estrogen levels (Figure 8). [31] There are number of sites of estrogen biosynthesis such as mesenchymal cells of adipose tissue and skin, [32] osteoblasts, [33] different region in the brain, [34] however ovaries are the main site for estrogen production in premenopausal nonpregnant women. The extragonadal sites of estrogen biosynthesis have several fundamental features differing from those of the ovaries and become major sources of estrogen beyond menopause. [32] In the present study female mice are comparable to the premenopausal stage and therefore ovaries are considered as primary source of the estrogen measured. Therefore based on the stage of the mice’s life cycle, length of the dietary treatment, we do not expect any major consequences due to the AT_2_R knock out status. However if HFD is continued on an aging mice population we could potentially see some additional worsening of the metabolic state in the mice.

While estrogen is a female hormone produced in higher quantity in females and known to regulate body weight gain, [24] there is evidence that male estrogen also plays a role in male body weight gain and influence fat metabolism. [35] Cells of the testes [36] produce estrogen, however testicular contribution for circulating estrogens at best is only 15% [37] making local production of estrogens, both intra testicular and extragonadal, physiologically significant. Nevertheless, since the levels of circulating estrogen in males compared to the females are so negligible in this study that in light of the large differential weight gain in females, actual site of estrogen biosynthesis in males does not seem very significant. There are evidences suggesting a positive regulatory interaction between female estrogen and AT_2_R; estrogen upregulates AT_2_R expression in the kidney, heart and reproductive organs. [15], [16], [25] Alternatively, stimulation of AT_2_R in ovarian granulose cells can induce ovulation and oocyte maturation, and increase estrogen production. [26] Consistent to these studies, we observed reduction in estrogen in AT_2_KO female mice in the present study. On the other hand, there are no reports on a potential interaction between male estrogens and AT_2_ receptor; however, we found that HFD caused a decrease in WT male urinary estrogen. The decrease was less pronounced in AT_2_KO male mice on HFD compared with WT on HFD, suggesting an inverse relationship between AT_2_R and male estrogen production.

Estrogen insufficiency is thought to be largely responsible for increased adiposity and abdominal fat gain during menopause. [9] Studies show post-menopausal women, ovariectomized mice, and mice lacking estrogen receptor-α or aromatase have increased fat mass, and develop obesity and hyperlipidemia. [4], [9], [35], [38] Additionally, physical inactivity has been observed in obese aromatase deficient rodents and in women transitioning to menopause. [4], [39] Based on our observations, we speculate that depletion of AT_2_R dysregulates estrogen level and thus function is compromised. Since adipose tissue also express both the AT_2_R and estrogen producing pathway, [8], [10], [40]–[41] absence of AT_2_R can cause a decrease in adipose estrogen production locally that could affect adipose tissue metabolism. Further increase in the body weight of AT_2_KO females when put on HFD may be due to compromised interaction between estrogen and insulin as well (Figure 2). It has been shown that chronic estrogen supplement in mice reduces the occurrence of HFD-induced insulin resistance, and improves glucose use rate. [24] In the present study, we demonstrated that HFD elicits profound reduction of estrogen level in female AT_2_KO that could have a negative effect on insulin and its function resulting in an increase in body weight and impaired glucose tolerance. These typical symptoms of metabolic syndrome may in turn further exacerbate weight gain due to compounding effect of the syndrome. As expected, the changes in plasma FFA and hepatic TG are aligned with changes in body weight gain, glucose tolerance and plasma insulin in WT and AT_2_KO male and females on HFD. Inasmuch as enhanced plasma FFA is indicative of impaired lipid metabolism, [42] plasma FAA also contributes to glucose intolerance and insulin resistance. [43], [44] Overall, the findings in this study indicate that differential changes in estrogen may be a potential basis of the gender bias role of AT_2_R in weight gain. Estrogen has also been implicated to regulate leptin an important hormone that regulates energy balance and food intake. [45], [46] There are studies suggesting that reduced estrogen associated weight gain and increased food intake may not be entirely associated with reduction in leptin. [9], [47] Moreover, since we did not observe any changes in calorie intake, (Figure 1) we speculate that affects of AT_2_R through estrogen on body weight gain may not involve leptin. However, the molecular mechanisms of AT_2_R-mediated regulation of estrogen in males and females, and in turn, the role of estrogen in AT_2_R-mediated weight gain need further investigation.

While estrogen could be a potential regulatory mediator in studying gender bias role of AT_2_R in weight gain as discussed above, other mechanisms cannot be ruled out. In previous studies, AT_2_R is suggested to promote adipocyte differentiation and lipogenesis. [48] Genes such as lipoprotein lipase, aP2 and CD36 proteins along with PPARγ are implicated in AT_2_R-mediated lipogenesis in adipocytes. [4], [49] While we have not measured these parameters, role of AT_2_R in lipogenesis in male mice may be attributed from our observation that there was a reduced adipose weight gain in AT_2_RKO mice on HFD. However, the results were quite opposite in females. There is study suggesting that estrogen or its metabolite may serve as an endogenous ligand for PPARγ [32] whose activation is known to improve insulin resistance and metabolism, [50]–[52] also seen in females in the present study. Whether such mechanisms make the basis for the gender biased role of AT_2_R in weight gain needs systematic investigation.

The present study provides a unique and novel perspective of the gender biased role of AT_2_R on body weight gain and other metabolic parameters. Differential regulation of estrogen by AT_2_R may be responsible in part for leading to gender bias body and fat weight gain and related effects such as glucose tolerance. However certain additions to the study would have provided a keener insight into the mechanistic aspects. These include metabolic and body composition analysis to determine the precise lipogenic/lipolytic factors contributing towards the weight gain. Effects of manipulation of estrogen by ovariectomy and estrogen replacement, globally as well as in metabolic tissues such as thyroid and fat, will be important to strengthen the AT_2_R-estrogen hypothesis. Additionally, along with the gender biased effect of AT_2_R, it would be interesting to know the effect of other RAS components such as AT_1_ receptor, angiotensin converting enzyme (ACE), ACE2 and renin, which are inter-regulatory. The present study paves way for future studies that could expand the scope of therapeutic potential of AT_2_R in metabolic disorders particularly in postmenopausal women.
